# Circulation of HDV Genotypes in Brazil: Identification of a Putative Novel HDV-8 Subgenotype

**DOI:** 10.1128/spectrum.03965-22

**Published:** 2023-04-19

**Authors:** Francisco C. A. Mello, Tairine M. Barros, Giovana P. Angelice, Vanessa D. Costa, Vinicius M. Mello, Maria Inês M. C. Pardini, Elisabeth Lampe, Barbara V. Lago, Livia M. Villar

**Affiliations:** a Laboratório de Hepatites Virais, Instituto Oswaldo Cruz, FIOCRUZ, Rio de Janeiro, Rio de Janeiro, Brazil; b Instituto de Tecnologia em Imunobiológicos (Bio-Manguinhos), FIOCRUZ, Rio de Janeiro, Rio de Janeiro, Brazil; c Universidade Estadual Paulista (Unesp), Faculdade de Medicina (FMB), Divisão Hemocentro, Laboratório de Biologia Molecular, Campus de Botucatu, Botucatu, São Paulo, Brazil; National Chung Hsing University

**Keywords:** HDV, genotype, HDV-8 subgenotype, Brazil

## Abstract

Hepatitis D virus (HDV) is classified into 8 genotypes (1 to 8) and several subgenotypes. In Brazil, HDV-3 and HDV-1 predominate; however, most of the diagnosis efforts and molecular studies are directed to the area of endemicity of the Amazon Basin. Here, we determined the molecular epidemiological profile of circulating HDV in Brazilian HBsAg-positive patients between 2013 and 2015 in areas of endemicity and non-areas of endemicity. From 38 anti-HDV-positive individuals, 13 (34.2%) had detectable HDV-RNA and 11 (28.9%) were successfully sequenced. Partial HDAg (~320 nt) sequencing followed by phylogenetic analysis with reference sequences resulted in the identification of HDV-3 (9/11; 81.8%), HDV-5 (1/11; 9.1%), and HDV-8 (1/11; 9.1%). Most HDV-3 samples (8/9; 88.9%) were found in the endemic North region, while one was found in Central-West Brazil, a non-area of endemicity. HDV-5 and 8, genotypes native from African countries, were found in São Paulo, a cosmopolitan city from Southeast Brazil with a high circulation of immigrants. Phylogenetic analysis of HDV-8 strains indicated that the sample determined in our study, along with previously reported sequences from Brazil, formed a highly supported monophyletic clade, likely representing a putative novel HDV-8 subgenotype.

**IMPORTANCE** Considered a neglected pathogen until the last 2 decades, an increase in the availability of genetic data of hepatitis D virus (HDV) strains around the world has been noticed recently, resulting in the proposition of different classifications. Our study aimed to determine the molecular epidemiological profile of HDV isolates circulating in areas of endemicity and non-areas of endemicity in Brazil. Based on the analyzed fragment, HDV-8 sequences clustered out of the clades formed by subgenotypes 8a and 8b might suggest the identification of a novel subgenotype, putatively designated subgenotype 8c. Our findings demonstrate the importance of continuous epidemiological surveillance to map HDV spread pathways and the introduction of imported variants. It also reinforces that as the amount of HDV genomes generated and reported increases, we will have changes in viral classification and, consequently, in our understanding of the dynamics of variability of this viral agent.

## INTRODUCTION

Hepatitis D virus (HDV) is a defective virus that requires hepatitis B virus (HBV) to replicate. Thus, hepatitis D is caused by the simultaneous infection of HBV and HDV (coinfection), or by secondary infection by HDV in HBV carriers (superinfection). HBV/HDV coinfection is the most severe form of viral hepatitis, often accelerating progression to cirrhosis and hepatocellular carcinoma (1).

It is estimated that 5% of HBV carriers are infected with HDV, resulting in 12 to 60 million seropositive individuals worldwide (2, 3). HDV endemicity varies according to geographic region. The Amazon Basin, in South America, is an important hot spot of high HDV prevalence (3, 4). In Brazil, 4,150 confirmed HDV cases were reported between 1999 and 2020 (5). A multicentric study conducted in all Brazilian regions revealed an overall HDV prevalence of 3.2%. Nevertheless, the distribution of HDV infection differed markedly in the distinct regions of the country, with North region, where the Amazon Basin is located, harboring 70% of positive cases (6).

Considered a neglected pathogen until the last 2 decades, an increase in the availability of genetic data of HDV strains around the world has been noticed recently, resulting in the proposition of different classifications for HDV (7–10). Based on genetic identity over the entire genome, HDV has been classified into 8 genotypes, numbered from 1 to 8, and several subgenotypes, with distinct distribution around the world. In Brazil, HDV-3 is the most prevalent and endemic to Amazon Basin (11, 12), followed by HDV-1, which is cosmopolitan (13, 14). Genotypes formerly considered to be native from specific areas of the globe, such as Africans HDV-5 and HDV-8, have already been reported in North and Northeast regions of Brazil, respectively (12, 15, 16), an expected trend considering the intense circulation of people around the globe and as studies on HDV infection grow worldwide.

Most studies conducted in Brazil are focused on areas of endemicity. However, a global understanding on HDV genetic variability is important to reach the World Health Organization (WHO) goal to eliminate viral hepatitis as a public health threat by 2030. This study aimed to determine the molecular epidemiological profile of HDV isolates circulating in areas of endemicity and non-areas of endemicity in Brazil.

## RESULTS

Of 38 anti-HDV positive samples from a previous survey (6) that were included in this study, 13 (13/38; 34.2%) had detectable HDV-RNA and 11 of them were successfully sequenced and genotyped (Table 1). Despite being HDV-RNA positive, two samples were not successfully sequenced likely due to low viral load.

**TABLE 1:** Characteristics of the samples according to their HDV-RNA status

	HDV RNA + *n* = 13 (%)	HDV RNA − *n* = 25 (%)	*P* value
Gender			0.73
Male	7 (53.8%)	11 (44.0%)	
Female	6 (46.2%)	14 (56.0%)	
Age			0.43
<20	1 (7.7%)	4 (16.0%)	
20–30	5 (38.5%)	0 (0%)	
31–40	1 (7.7%)	4 (16.0%)	
41–50	1 (7.7%)	9 (36.0%)	
51–60	2 (15.4%)	5 (20.0%)	
>60	1 (7.7%)	0 (0%)	
Missing	2 (15.4%)	3 (12.0%)	
Brazilian regions			0.76
North	8 (61.5%)	19 (76.0%)	
Northeast	1 (7.7%)	1 (4.0%)	
Central-west	1 (7.7%)	2 (8.0%)	
Southeast	3 (23.1%)	3 (12.0%)	
South	-	-	

**FIG 1:**
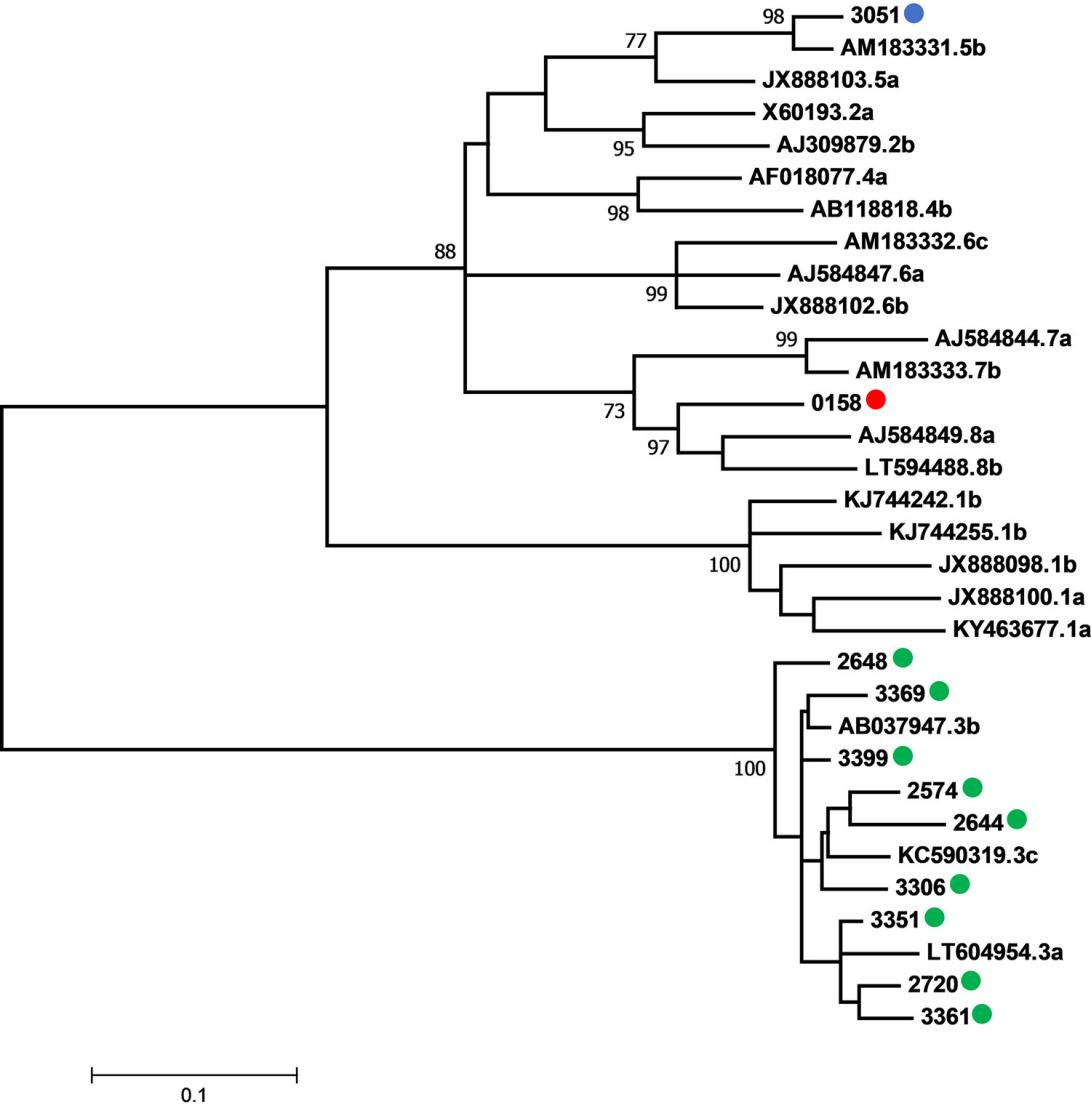
Maximum likelihood tree constructed under HKY + G+I model for definition of HDV genotypes. HDV-3 sequences determined in this study are represented by green circles, grouped with reference sequences from Bolívia (LT604954), Brazil (KC590319) and Venezuela (KC590319). HDV-5 sequence determined in this study is represented by a blue circle, grouped with a reference sequence from Guinea-Bissau (AM183331). HDV-8 sequence obtained here is represented by a red circle, grouped with reference sequences from the Republic of the Congo (AJ584849 and LT594488). Reference sequences are represented by their accession number in GenBank followed by the genotype classification as proposed by Miao et al. (2019) (10). Bootstrap values higher than 70% are represented in the nodes of the tree.

Phylogenetic analysis indicated the circulation of HDV-3 in 9/11 (81.8%) samples, eight from the endemic North Region where the Amazon Basin is located, and one from the state of Mato Grosso do Sul, located in the Central-West region (non-area of endemicity), which borders the Amazon Basin. The other two samples were obtained from individuals living in the state of São Paulo, Southeast region (non-area of endemicity), and were classified as HDV-5 (1/11; 9.1%) and HDV-8 (1/11; 9.1%) (Fig. 1).

Intragenotype genetic distance within HDV-3 sequences considering the 320-nt fragment analyzed here was 0.016 ± 0.003. This high genetic similarity of HDV-3 sequences prevented the formation of well-defined clusters distinguishing subgenotypes 3a, 3b, and 3c.

Phylogenetic analysis performed on a genotype-specific data set composed of HDV-5 sequences indicated that the HDV-5 sequence characterized here clustered with sequences classified as subgenotype 5b, presenting high similarity with a sequence from Guiné-Bissau (tree not shown). The genetic distance between the group of sequences characterized as HDV-5a and HDV-5b was 0.085 ± 0.011 while the intragroup distance was 0.064 ± 0.009 for HDV-5a sequences and 0.059 ± 0.009 for HDV-5b.

**FIG 2:**
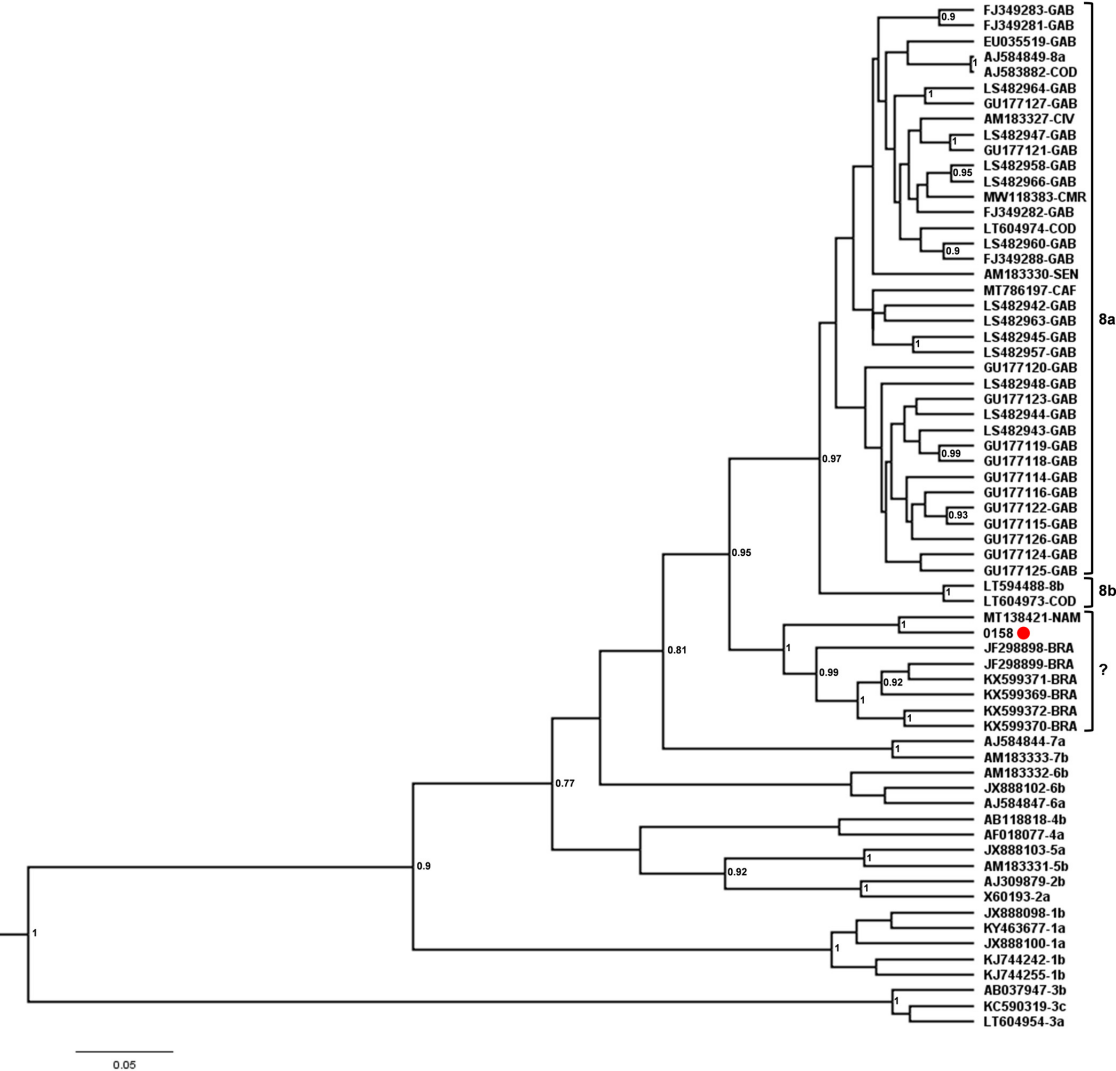
Evidence for a distinct lineage among HBV-8 isolates. Phylogenetic trees incorporated 46 HDV-8 isolates whose partial HDAg nucleotide sequences were available in GenBank along with the sequence described in this study, highlighted with a red circle. Reference sequences classified as HDV-8 are represented by their accession number in GenBank followed by the abbreviation of its country of origin according to ISO 3166 code. The reference sequences of the remaining genotypes were represented by the accession number, followed by the genotype classification as proposed by Miao et al. (2019) (10). Phylogenetic analysis was performed by Bayesian Inference using the Bayesian Markov chain Monte Carlo (MCMC) statistical framework. The putative novel HDV-8 subgenotype monophyletic clade, shown in square brackets with a question mark, was established with a posterior probability = 1.

**FIG 3:**
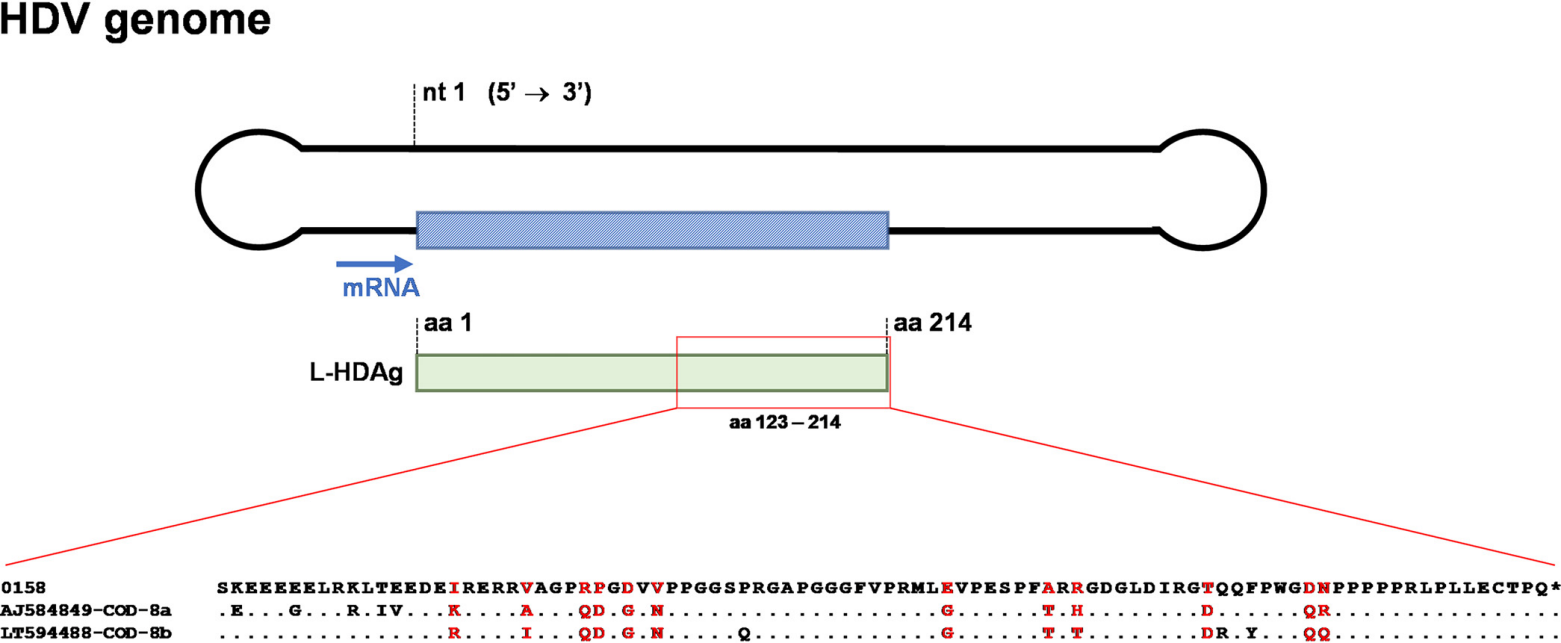
Amino acid alignment of the l-HDAg fragment (aa 123–214) containing reference sequences of HDV subgenotype 8a (AJ584849), 8b (LT594488), and the sequence determined in the current study (0158) that clustered in the putative HDV-8c monophyletic clade. Amino acid residues exclusively found in the determined sequence are shown in red.

Regarding HDV-8, our sequence was closely related to a sequence from Namibia and clustered in a distinct monophyletic clade together with Brazilian sequences from Maranhão, a state located in the Northeast region of Brazil (15, 16) (Fig. 2). Based on the analyzed fragment, these sequences clustered out of the clades formed by subgenotypes 8a and 8b might be suggesting the identification of a novel subgenotype, putatively designated subgenotype 8c. Genetic distance among the proposed subgenotype 8c sequences and the subgenotypes 8a and 8b was 0.149 ± 0.017 and 0.144 ± 0.018, respectively, while the genetic distance observed between subgenotypes 8a and 8b was 0.083 ± 0.015. Fig. 3 illustrates the partial portion of l-HDAg analyzed and the comparison of deduced amino acid sequences of HDV subgenotypes 8a, 8b, and the putative subgenotype 8c.

## DISCUSSION

This study describes the circulation of three HDV genotypes (HDV-3, HDV-5, and HDV-8) in Brazil, in both areas of endemicity (North region) and non-areas of endemicity (Central-West and Southeast regions) (Fig. S1). Surveillance of hepatitis D has been restricted to chronic HBV carriers living in the North Region, with few reports from states outside the Amazon Basin. Nevertheless, knowing the scope of HDV infection in Brazil and accessing the genetic diversity of this virus inside and outside regions of endemicity can provide useful information to contextualize Brazil in face of the goal proposed by WHO to eliminate viral hepatitis by 2030. Furthermore, HDV molecular characterization is a useful tool for detecting the introduction and circulation of imported genotypes as well as the most plausible routes of transmission throughout the country.

Previous reports have shown that HDV-3 is the most prevalent genotype in Brazil, with a minor presence of HDV-1 (11, 12, 15–18). HDV-3 is the main genotype found in South America and its circulation is restricted to the American continent, whereas HDV-1 is found worldwide (13, 14).

Here, no HDV-1 was found among the 11 HDV-RNA positive samples. HDV-3 was described in 9/11 (81.8%) samples, all but one from individuals living in the endemic North region. The remaining HDV-3 isolate was from an individual from the state of Mato Grosso do Sul, Central-West region. Even considered a non-region of endemicity, Central-West borders four states in the North region, which makes viral dissemination more feasible.

Despite HDV-3 being the most divergent of all HDV genotypes, it presents a restricted intragroup genetic variability (8, 10). Considering the 320-nt fragment analyzed here, intragenotype genetic distance was 0.016 ± 0.003. The low genetic diversity together with the fact that HDV-3 is virtually restricted to South America support the hypothesis of a recent origin, as suggested by Nogueira-Lima and colleagues (2019) (19). Geographic isolation of native tribes and difficult access to the inner Amazon Basin may play a role in the high prevalence of HDV infection in this area and in its low genetic diversity (18). In contrast, the geographic distribution of other HDV genotypes has changed over time globally, mainly due to immigration waves from countries of endemicity (20).

Genotypes HDV-5 and HDV-8, usually found in Africa (9, 21, 22), were detected in two individuals from São Paulo, the most populated Brazilian city and the main destination for immigrants from different parts of the world. Both HDV-5 and HDV-8 carriers had typical African names and surnames, likely indicating their origin and that an allochthonous infection might have occurred. However, due to the lack of socio-demographic data, this assumption could not be confirmed.

HDV-5 has been previously reported in Cameroon, Mauritania, and Nigeria (13). However, recent studies identified this genotype in Switzerland, the United Kingdom (13), and Australia (23). In Brazil, HDV-5 had already been reported in two individuals from the North region (12); however, nucleotide sequences of such samples were not available in the GenBank database, preventing them from being included in our analyses. Here, the HDV-5 sample was closely related to a sequence from Guinea-Bissau, located in West Africa (22).

HDV-8 has been described in Gabon, Congo, Ivory Coast, and Senegal (13, 22, 24). In the last decade, two studies carried out in the state of Maranhão, Northeast Brazil, identified the circulation of HDV-8 (15, 16). These findings led the authors to suggest a link to the slave trade that occurred from the 16^th^ to the mid-19^th^ century. In our study, analysis of partial HDV-8 genome demonstrated the existence of a highly supported separated monophyletic clade, composed by other Brazilian HDV-8 sequences and a sequence from Namibia, apart from subgenotypes 8a and 8b. The genetic distance between sequences from this clade and HDV-8a or HDV-8b sequences was higher than that observed between subgenotypes 8a and 8b (0.14 versus 0.08). This finding suggests that this new clade of HDV-8 sequences could be classified in a new subgenotype, which we would putatively designate as 8c. Phylogenetic trees reconstructed by phylogeny software based on the maximum-likelihood principle (IQ-TREE v.1.6.12) employing two statistical support methods, aLRT and bootstrap analysis with 1,000 replicates, also supported the distinct clade with values of 87.4 and 92, respectively (Fig. S2). Further phylogenetic and phylogeographic analysis comprising whole HDV genome and gathering previous information on sample collection date and locality would confirm or not this hypothesis.

This study had some limitations. The main limitation consisted of a phylogenetic analysis based on a fragment of 320 bp of the viral genome, although a recent study on HDV classification reported no disagreement between analyses encompassing whole-genome sequences or partial l-HDAg encoding sequences (8). In addition, the absence of socio-demographic data hindered determination of whether the imported genotypes came from Brazilian or foreign individuals.

Despite these limitations, our findings demonstrate the importance of continuous epidemiological surveillance to map HDV spread pathways and the introduction of imported variants. It also reinforces that as the amount of HDV genomes generated and reported increases, we will have changes in viral classification and, consequently, in our understanding of the dynamics of variability of this viral agent. Identification of new strains and their dissemination in places where HDV diagnosis is not routinely performed might contribute to the implementation of effective measures to control HDV infection in these areas.

## MATERIALS AND METHODS

This study was performed using 38 anti-HDV positive serum samples, obtained from a previous sampling of 1,240 HBsAg-positive individuals from nearly all Brazilian states, collected from 2013 to 2015 (6) and stored in a biorepository. The present study was approved by the Ethical Committee of the Oswaldo Cruz Foundation (FIOCRUZ) under CAAE number 77364217.0.0000.5248, that is in accordance with the Declaration of Helsinki.

In order to detect HDV-RNA, nucleic acid was extracted from 200 μL HDV-antibody-positive serum using “High Pure Viral Nucleic Acid kit” (Roche Life Science, Mannheim, Germany), according to the manufacturer’s instructions. Partial hepatitis delta antigen (HDAg) amplification (~320 bp) was performed by RT-PCR using “Superscript III One-Step RT-PCR system” (Thermo Fisher Scientific, Waltham, USA) followed by a nested PCR as previously described (11). DNA sequencing was performed with “BigDye Terminator Cycle Sequencing 3.1” (Applied Biosystems, CA, USA) using the same primer pair from the second PCR. Sequences were assembled, aligned using the MUSCLE algorithm and analyzed with MEGA software version X (25) to determine the genotypes and to access the HDV genetic diversity.

Genotyping was performed through phylogenetic comparison employing a data set composed of 21 representative sequences of all HDV subgenotypes as suggested by Miao et al., 2019 (10). Phylogenetic analyses were conducted using the Maximum Likelihood method, under the HKY+G+I substitution model as the best-fit model, with a 1,000 replicate bootstrap resampling. Online tool BLAST (“Basic Local Alignment Search Tool”) was accessed to create a genotype-specific data set containing highest genetic similarity sequences of HDV-3 (*n* = 65), HDV-5 (*n* = 16), and HDV-8 (*n* = 18), which were submitted to a new phylogenetic evaluation with a Bayesian Inference using the Bayesian Markov chain Monte Carlo (MCMC) statistical framework implemented in BEAST v1.10.4 software.

As HDV-8 sequences available in GenBank were shorter than the sequence determined here, the multiple sequence alignment was shortened to 293 nt prior to phylogenetic analysis. Intragenotypic and intersubgenotypic diversity was evaluated by genetic distance calculation using MEGA X software (25). Nucleotide sequences obtained during this study were deposited in the GenBank database under accession numbers ON286983-ON286993. Information regarding the sequences used in the analyses are available in the supplementary table.

Clinical and epidemiological data were coded and entered into a datasheet (Excel 2010, Microsoft Inc., USA). Descriptive statistics were generated for the responses, and chi-squared test for independence or for trend and Fisher Exact Test were used to compare categorical variables according to HDV-RNA positivity in GraphPad InStat. *P* value < 0.05 was considered statistically significant.

The study was conducted in accordance with the Declaration of Helsinki and approved by the Institutional Ethics Committee of Oswaldo Cruz Foundation (FIOCRUZ) under CAAE number 77364217.0.0000.5248.

Informed consent was obtained from all subjects involved in the study.

### Data availability.

Nucleotide sequences obtained during this study were deposited in the GenBank database under accession numbers ON286983-ON286993. Information regarding the sequences used in the analyses are available in supplementary table.
