# Supplementary material for: Circulation of HDV Genotypes in Brazil: Identification of a Putative Novel HDV-8 Subgenotype
Source: Microbiol Spectr. 2023 Apr 19;11(3):e03965-22. doi: 10.1128/spectrum.03965-22 (PMC10269522; doi:10.1128/spectrum.03965-22)
Supplement: Supplemental file 1 — Table S1 and Fig. S1 and S2. Download spectrum.03965-22-s0001.pdf, PDF file, 0.4 MB [file spectrum.03965-22-s0001.pdf]

Table S1. List of the HDV nucleotide sequences (Genbank Accession numbers) used in phylogenetic analysis classified by genotype and country of origin.

Accession number	Genotype	Country
AM183331	5b	Guinea-Bissau
JX888103	5a	Nigeria
X60193	2a	Japan
AJ309879	2b	Russia
AF018077	4a	Taiwan
AB118818	4b	Japan
AM183332	6c	Central African Republic
AJ584847	6a	Cameroon
JX888102	6b	Nigeria
AJ584844	7a	Cameroon
AM183333	7b	Cameroon
AJ584849	8a	Democratic Republic of the Congo
LT594488	8b	Democratic Republic of the Congo
KJ744242	1b	Iran
KJ744255	1b	Iran
JX888098	1b	Nigeria
JX888100	1a	Nigeria
KY463677	1a	Ethiopia
AB037947	3b	Venezuela
KC590319	3c	Brazil
LT604954	3a	Bolivia
FJ349283	8a	Gabon
FJ349281	8a	Gabon
EU035519	8a	Gabon
AJ584849	8a	Democratic Republic of the Congo
AJ583882	8a	Democratic Republic of the Congo
LS482964	8a	Gabon
GU177127	8a	Gabon
AM183327	8a	Cote d'Ivoire
LS482947	8a	Gabon
GU177121	8a	Gabon
LS482958	8a	Gabon
LS482966	8a	Gabon
MW118383	8a	Cameroon
FJ349282	8a	Gabon
LT604974	8a	Democratic Republic of the Congo
LS482960	8a	Gabon
FJ349288	8a	Gabon
AM183330	8a	Senegal
MT786197	8a	Central African Republic
LS482942	8a	Gabon
LS482963	8a	Gabon
LS482945	8a	Gabon
LS482957	8a	Gabon
GU177120	8a	Gabon
LS482948	8a	Gabon

GU177123	8a	Gabon
LS482944	8a	Gabon
LS482943	8a	Gabon
GU177119	8a	Gabon
GU177118	8a	Gabon
GU177114	8a	Gabon
GU177116	8a	Gabon
GU177122	8a	Gabon
GU177115	8a	Gabon
GU177126	8a	Gabon
GU177124	8a	Gabon
GU177125	8a	Gabon
LT594488	8b	Democratic Republic of the Congo
LT604973	8b	Democratic Republic of the Congo
MT138421	8?	Namibia
JF298898	8?	Brazil
JF298899	8?	Brazil
KX599371	8?	Brazil
KX599369	8?	Brazil
KX599372	8?	Brazil
KX599370	8?	Brazil
AJ584844	7a	Cameroon
AM183333	7b	Cameroon
AM183332	6b	Central African Republic
JX888102	6b	Nigeria
AJ584847	6a	Cameroon
AB118818	4b	Japan
AF018077	4a	Taiwan
JX888103	5a	Nigeria
AM183331	5b	Guinea-Bissau
AJ309879	2b	Russia
X60193	2a	Japan
JX888098	1b	Nigeria
KY463677	1a	Ethiopia
JX888100	1a	Nigeria
KJ744242	1b	Iran
KJ744255	1b	Iran
AB037947	3b	Venezuela
KC590319	3c	Brazil
LT604954	3a	Bolivia

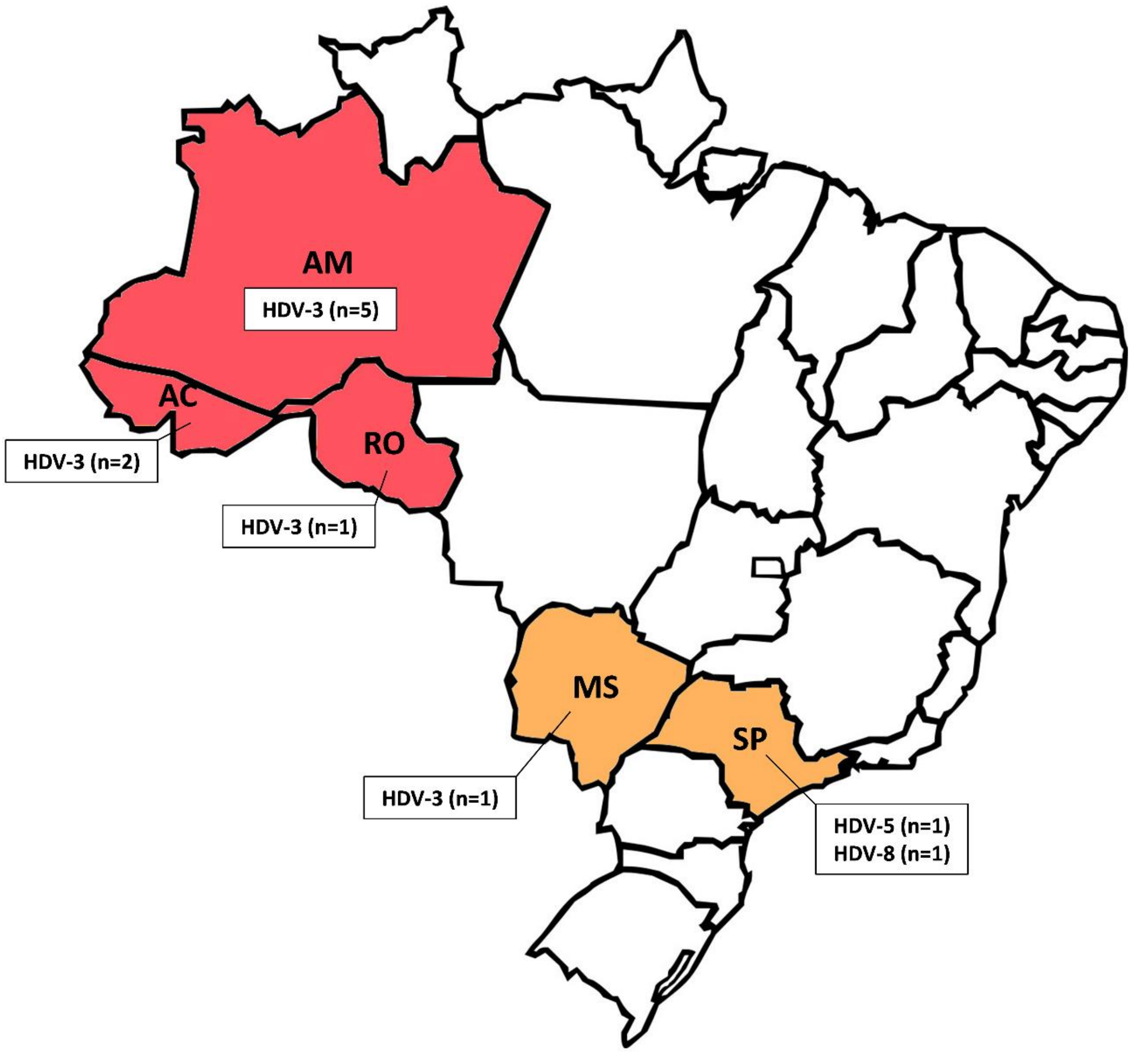

Figure S1. Map of Brazil indicating from which states were HDV-RNA positive samples collected (AM: Amazonas; AC: Acre; RO: Rondônia; MS: Mato Grosso do Sul; SP: São Paulo). States located in endemic areas for HDV infection are represented in red whereas states located in non-endemic areas are represented in orange.

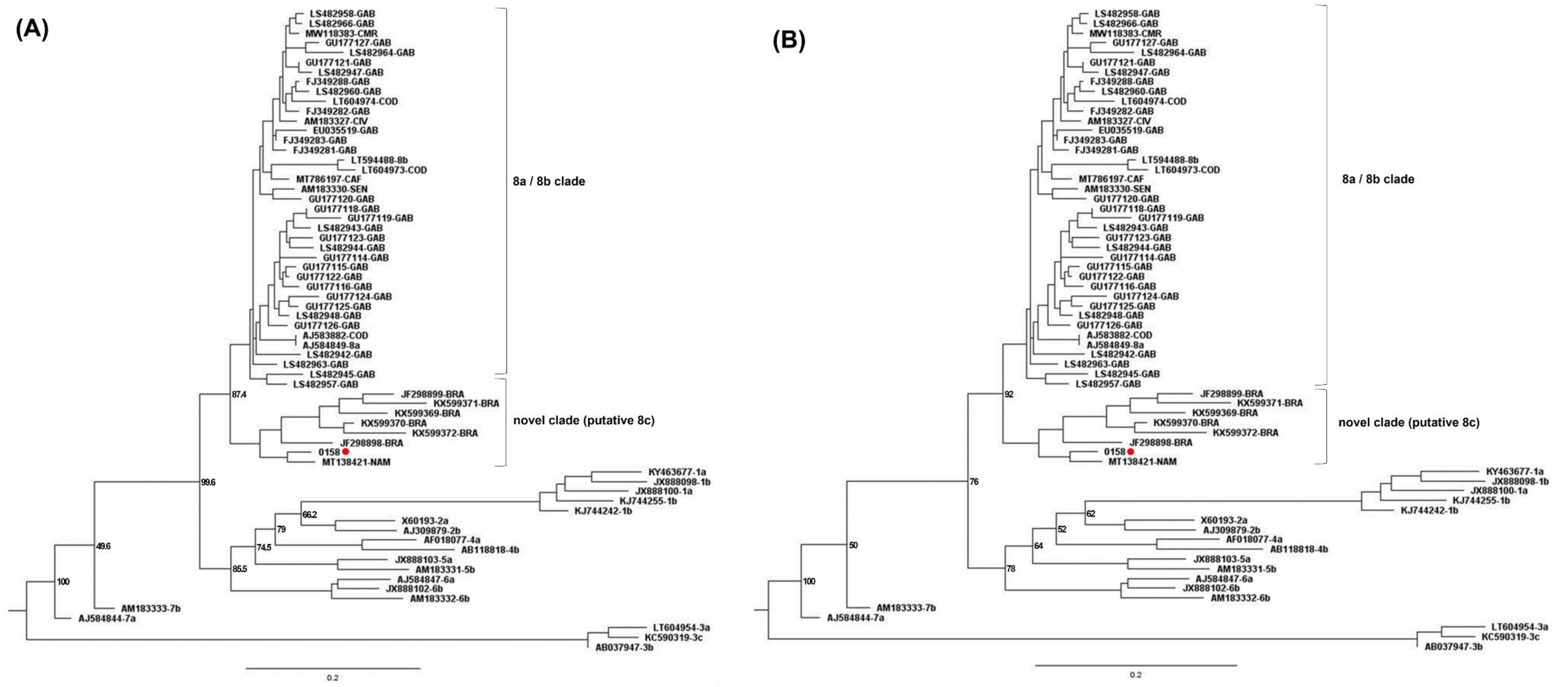

Figure S2. Maximum-likelihood phylogenetic trees employing two statistical support methods, aLRT (A) and bootstrap analysis (B) with 1000 replicates. The putative novel HDV-8 subgenotype monophyletic clade containing the sequence described in this study (0158), represented with a red circle, was highly supported by both statistical methods.
